# Prevalence and antimicrobial resistance profile of bacterial foodborne pathogens in Nile tilapia fish (*Oreochromis niloticus*) at points of retail sale in Nairobi, Kenya

**DOI:** 10.3389/frabi.2023.1156258

**Published:** 2023-05-24

**Authors:** Millicent T. Mumbo, Evans N. Nyaboga, Johnson Kinyua, Edward K. Muge, Scholastica G. K. Mathenge, Geoffrey Muriira, Henry Rotich, Bernard Njiraini, Joshua M. Njiru

**Affiliations:** ^1^ Department of Biochemistry, Jomo Kenyatta University of Agriculture and Technology, Nairobi, Kenya; ^2^ Department of Biochemistry, University of Nairobi, Nairobi, Kenya; ^3^ Department of Medical Laboratory Science, Kenyatta University, Nairobi, Kenya; ^4^ Research and Development Department, Kenya Bureau of Standards, Nairobi, Kenya

**Keywords:** antibiotic resistance, fishborne bacteria, multidrug resistance, *Oreochromis niloticus*, prevalence

## Abstract

*Proteus spp*., *Staphylococcus spp*., *Pseudeomonas spp*., and pathogenic Vibrios are among the major foodborne pathogens associated with the consumption of contaminated fish. The increasing occurrence of antimicrobial resistance in these pathogens is a serious public health concern globally and therefore continuous monitoring of antimicrobial resistance of these bacteria along the food chain is crucial for for control of foodborne illnesses. The aim of this study was to assess the prevalence, antimicrobial resistance patterns, antibiotic resistance genes, and genetic diversity of bacterial foodborne pathogens recovered from fresh Nile tilapia (*Oreochromis niloticus*) obtained from retail markets in Nairobi, Kenya. A total of 68 *O. niloticus* fish with an average weight of 300.12 ± 25.66 g and body length of 23.00 ± 0.82 cm were randomly sampled from retail markets and tested for the presence of *Proteus, Staphylococcus aureus, Pseudomonas aeruginosa, Vibrio cholerae*, and *Vibrio parahaemolyticus*. Standard culture-based microbiological and Kirby–Bauer agar disk diffusion methods were used to isolate and determine the antimicrobial resistance patterns of the isolates to 11 selected antibiotics. Statistical analysis was performed using Minitab v17.1, with *p* < 0.05 considered significant. The genetic diversity of the multidrug-resistant (MDR) and extensively drug-resistant (XDR) bacteria was determined using 16S rRNA sequencing and phylogenetic analysis, and polymerase chain reaction (PCR) was used for detection of antibiotic resistance genes in MDR bacterial isolates. High levels of bacterial contamination were detected in fresh *O. niloticus* fish (44/68, 64.71%). The most prevalent bacteria were *Proteus* spp. (44.12%), with the rest of the bacterial species registering a prevalence of 10.29%, 4.41%, 2.94%, and 2.94% (for *S. aureus*, *P. aeruginosa*, *V. cholerae*, and *V. parahaemolyticus*, respectively). Antimicrobial resistance was detected in all the bacteria species and all the isolates were resistant to at least one antibiotic except cefepime (30 µg). Additionally, 86.36% of the isolates exhibited multidrug resistance, with higher multiple antibiotic resistance indices (MAR index >0.3) indicating that fresh *O. niloticus* fish were highly contaminated with MDR bacteria. Results of 16S rRNA sequences, BLASTn analysis, and phylogenetic trees confirmed the identified MDR bacterial isolates as *Proteus mirabilis* and other *Proteus* spp.*, S. aureus*, *P. aeruginosa*, *V. cholerae*, and *V. parahaemolyticus*. PCR analysis confirmed the presence of multiple antibiotic resistance genes *bla*TEM-1, *bla*CMY-2, *tet*A, *tet*C, *Sul*2, *dfr*A7, *str*A, and *aad*A belonging to β-lactamases, tetracycline, sulfonamide, trimethoprim, and aminoglycosides in all the MDR bacterial isolates. There was strong correlation between antibiotic- resistant genes and phenotypic resistance to antibiotics of MDR bacteria. This study showed high prevalence of multidrug resistance among foodborne bacterial isolates from fresh *O. niloticus* fish obtained from retail markets. From this study, we conclude that fresh *O. niloticus* fish are a potential source of MDR bacteria, which could be a major risk to public health as a consequence of their dissemination along the human food chain. These results highlight the prevalence of antimicrobial-resistant foodborne pathogens in fish purchased from retail markets and underscore the risk associated with improper handling of fish.

## Introduction

Bacterial contamination is responsible for more than 600 million cases of foodborne illnesses, with 420,000 fatal infections annually (World Health Organization, 2015). This is a huge economic burden to low- and middle-income countries because the estimated cost of treatment is about USD 110 billion per year. The main cause of foodborne infections is the presence of pathogenic bacteria which produce toxins in foods, especially animal-derived foods (Addis and Sisay, 2015). Fish has been identified as one of the reservoirs of pathogenic bacteria linked to human illnesses (Novotny et al., 2004). Fish is an integral part of the human diet for many generations and is promoted because of its health benefits, providing a rich source of animal protein, omega-3 fatty acids, vitamin D, selenium, and iodine (Tørris et al., 2018). Of particular interest is Nile tilapia (*Oreochromis niloticus*), which has become more popular as indicated by increasing levels of consumption (Obiero et al., 2022). However, both raw and undercooked fish can expose consumers to many types of pathogenic bacteria, either from their original aquatic environment or their postharvest handling, storage, and processing conditions (Vázquez-Sánchez et al., 2012; Baliere et al., 2015). Therefore, pathogenic bacteria can be introduced into fish at any point throughout the production and supply chain.

Foodborne pathogens such as *Plesiomonas shigelloides*, *Salmonella* spp., *Aeromonas* spp., *Proteus* spp., *Yersinia*, *Shigella*, *Enterobacter*, *P. aeruginosa*, *S. aureus*, *V. parahaemolyticus*, *V. cholerae*, *Bacillus*, *Klebsiella*, *Serratia* spp., and pathogenic *Escherichia*, are of importance in fish (Novotny et al., 2004) because they are responsible for foodborne illnesses such as diarrhea, gastroenteritis, typhoid fever, and dysentery. These illnesses pose significant health risks, including death, to consumers (Obande et al., 2017). Outbreaks of fish-associated food poisoning are caused by the consumption of raw or insufficiently heat-treated fish contaminated with *Vibrios* from the water environment (*Vibrio* spp.) or terrestrial sources (*Salmonella* spp., *Shigella* spp., *Staphylococcus* spp., *Pseudomonas* spp.) (Novotny et al., 2004). *Vibrios* are Gram-negative bacteria and ubiquitous in aquatic environments such as aquaculture, marine, and estuarine, either free-living in water, sediments or associated with shrimps and fishes (Vezzulli et al., 2010). Pathogenic *Vibrios* of major public health importance are *Vibrio vulnificus*, *V. parahaemolyticus*, and *V. cholerae*. *Vibrios* species have been observed to be associated with deadly cholera outbreaks globally (WHO, 2021a). For example, the consumption of dried fish was linked with a cholera epidemic in a village (Ifakara) in southern Tanzania in 1997 (Acosta et al., 2001). An outbreak of cholera in Germany in 2001 was associated with fresh fish imported from Nigeria (Schurmann et al., 2002). Therefore, continuous monitoring of *Vibrios* in fish is important for food safety control.

*S. aureus*, a Gram-positive bacterium, is the most prevalent foodborne pathogen of the genus *Staphylococcus* and among the leading causes of food contamination, and spoils food by producing lethal enterotoxin (Akbar and Anal, 2014). *S. aureus* has been reported in fish and fishery products (Vaiyapuri et al., 2019). *S. aureus* is not a natural microbiota of fish and therefore its presence in fish is associated with unhygienic handling by fish handlers, processors or sellers, cross-contamination during transport and storage, and contamination by workers, due to the presence of this pathogen in the microbiome of most humans (Saito et al., 2011; Hammad et al., 2012; Sergelidis et al., 2014; Badawy et al., 2022). *Proteus mirabilis* is a Gram-negative, rod-shaped bacterium found in the environment, animal microbiota and humans (Drzewiecka, 2016), and an important zoonotic pathogen that causes infections in animals and humans. It causes human urinary tract infections and extraintestinal infections such as respiratory, ear, eye, nose, skin, and wound infections (Sanches et al., 2019). *P. mirabilis* causes food poisoning through consumption of contaminated food, and high incidence has been reported in various countries (Gong et al., 2019). Therefore, presence of *P. mirabilis* in fish is a threat to the health of the consumer, especially when the specific strain possesses a variety of virulence factors that contribute to human infections. *P. aeruginosa* is an opportunistic bacterium with the ability to inhabit animals, soil, water, and plants, from which it is easily transmissible. It is one of the major causes of bacterial diseases in fish and there is growing evidence of *P. aeruginosa* in foodborne infections (Bricha et al., 2009; Nawaz and Bhattarai, 2015; Virupakshaiah and Hemalata, 2016). Various *Pseudomonas* species are multidrug resistant and resistance is likely to evolve over time, which explains why the number of effective antibiotics is decreasing and this may pose threat to public health (Algammal et al., 2020; Ayman et al., 2022). It is therefore important to determine the role of *O. niloticus* fish as a reservoir of *P. aeruginosa*.

Bacterial pathogens are among the priority microbial fishborne hazards, and the carriage of antimicrobial resistance (AMR) by these bacteria or other fish microbiota adds a further dimension to their importance. According to the World Health Organization (WHO), antimicrobial resistance is among the top 10 global public health threats (WHO, 2021b). The development and spread of AMR is propagated by the use of antibiotics for growth promotion and prophylactic measures. Regular monitoring and surveillance of antibiotic-resistant bacteria that contaminate foods may help to track the cause of foodborne diseases and lead to appropriate safety policy for interventions, prevention and/or effective treatment of foodborne diseases. Bacteria species belonging to the *Enterobacteriaceae* family are included in AMR surveillance programmes worldwide. Previous studies have shown that foodborne pathogens isolated from fish could be resistant to various antibiotics, including methicillin (Sergelidis et al., 2014). Fish may potentially facilitate the spread of antimicrobial resistance determinants to other bacteria species through horizontal gene transfer, thus making them a threat to public health and safety (Walsh et al., 2008).

Multidrug resistance (MDR) has increased all over the world and is considered a public health threat (Catalano et al., 2022). Several recent investigations reported the emergence of multidrug-resistant bacterial pathogens from different origins, which increases the necessity of the proper use of antibiotics, routine applications of the antimicrobial susceptibility testing to detect the antibiotic of choice, and screening of emerging MDR strains (Abolghait et al., 2020; Algammal et al., 2020; Algammal et al., 2022). MDR in bacteria may be generated by several mechanisms. First, bacteria may accumulate multiple genes—each coding for resistance to a single drug—within a single cell, and this accumulation typically occurs on resistance (R) plasmids. Moreover, multidrug resistance may also occur due to the increased expression of genes that code for multidrug efflux pumps, extruding a wide range of drugs (Catalano et al., 2022). Finally, MDR can be developed by enzymatic inactivation of the drugs through their degradation or by transfer of a chemical group to them (Reygaert, 2018). Microbial virulence factors are molecules produced by pathogenic microorganisms and have the ability to evade their host defenses and cause disease. These molecules range from secreted products such as enzymes, toxins, and exopolysaccharides, to cell surface structures such as lipopolysaccharides, capsules, lipoproteins, and glycoproteins. Some secreted molecules can manipulate the host cell machinery and hence cause infection (Jorge, 2020).

Given the widespread consumption of *O. niloticus* in urban communities in Kenya, there is a need to conduct a surveillance study to determine the safety of *O. niloticus* consumed. To the best of our knowledge, this is the first study to evaluate the bacterial contamination of *O. niloticus* fish in Kenya with emphasis on *Proteus* spp., *S. aureus*, *P. aeruginosa*, *V. cholerae*, and *V. parahaemolyticus*. Therefore, this study aimed to determine the prevalence of foodborne bacterial pathogens in retailed fresh *O. niloticus* fish in markets in Nairobi, Kenya. It also examined the antibiotic resistance of the isolated bacteria species and determined the genetic diversity of MDR bacteria. The findings provide prerequisite information supporting the need for control and prevention of outbreaks associated with exposure to the pathogenic bacteria in Kenya.

## Materials and methods

### Study site and sample collection

This study was conducted in fresh retail markets of Nairobi County, Kenya. Fresh *O. niloticus* fish samples (n = 68) were collected from markets in five sub-counties, namely Kasarani (n = 14), Makadara (n = 14), Westlands (n = 14), Embakasi (n = 13) and Lang’ata (n = 13). The *O. niloticus* fish samples were randomly collected from different retail markets within the five sub-counties. The fish samples were collected and packaged by the retailer in sterile, transparent, ziplock polypropylene bags and were transported in ice boxes to the microbiology laboratory at the Department of Biochemistry, University of Nairobi, within 1 hour of purchase, and analysis was conducted within 3 hours of collection.

### Isolation and identification of bacterial pathogens

*O. niloticus* fish samples were aseptically dissected to obtain tissue samples (flesh and gills) and were prepared for bacteriological examination according to the ISO 6887-3:2003 standard (ISO standard 6887, 2003). The samples were homogenized and subjected to analysis of *Proteus* spp., *S. aureus*, *P. aeruginosa*, *V. cholerae*, and *V. parahaemolyticus*.

*Vibrio* spp.: The homogenate was inoculated in 6 ml of alkaline peptone water (APW), pH 8.6, for enrichment, and incubated at 37°C for 8 hours. Two loopfuls of APW from the surface and topmost portion of the broth were inoculated on CHROMagar™ Vibrio (CHROMagar, Paris, France). Characteristic green-blue to turquoise blue (*V. cholerae*) and mauve colonies (*V. parahaemolyticus*) were picked and purified on thiosulfate-citrate-bile-salts-sucrose agar (Oxoid, Thermo Fischer Scientific, Lenexa, United States) with incubation at 37°C for 2 hours. The suspected colonies were re-streaked on tryptone soy agar (Oxoid, Thermo Fischer Scientific) supplemented with 3% sodium chloride (TSA + 3% NaCl) and incubated at 37°C for 2 hours to obtain pure isolates. The suspected *V. parahaemolyticus* colonies were confirmed by API 32E and VITEK 2 Compact, and later by halotolerance test with different concentrations of NaCl.

*S. aureus*: The homogenate was inoculated on Baird-Parker agar and incubated at 37°C for 48 hours. *Staphylococcus* isolates were detected using the ISO6888-3:2003 + AC: 2005 method (ISO Standard 6888, 2003). Characteristic gray-black colonies with a halo were picked and sub-cultured on mannitol salt agar plates to obtain pure cultures. The coagulase-positive colonies obtained were then confirmed using API Staph and VITEK 2 Compact for Gram-positive bacteria.

*Proteus* spp.: A 1 ml aliquot of the homogenate was inoculated on blood agar (HiMedia, Mumbai, India) and incubated at 28°C for 24 hours. The colonies were inoculated into 5 ml tryptose soya broth (TSB; HiMedia, Mumbai, India) in falcon tubes and incubated at 27°C for 24 hours. The inocula were streaked on xylose lysine deoxycholate Agar (XLD) and MacConkey agar (HiMedia, Mumbai, India) plates. The presumptive colonies of *Proteus* spp. with black colonies on XLD and pale or colorless colonies on MacConkey agar were selected.

*P. aeruginosa*: A 1 ml homogenate was inoculated in nutrient broth for enrichment and incubated at 37°C for 24 hours followed by culturing on pseudomonas cetrimide agar (PCA) (Labobasi, Mendrisio, Switzerland) plates (as selective media culture) and incubated at 37°C for 24 hours. Green-blue-pigmented colonies were considered colonies suspected of containing the *Pseudomonas* genus. Biochemical tests, including fermentation of lactose, indole, citrate, and oxidase, and hemolysis in blood, were performed to confirm *P. aeruginosa*. Colonies containing lactose-negative, citrate-positive, indole-negative, oxidase-positive and hemolytic bacteria were identified as *P. aeruginosa*.

### Antimicrobial resistance testing

The antimicrobial resistance test was performed using the Kirby–Bauer disk diffusion method (Mohamed et al., 2019) according to the guidelines of the Clinical Laboratory Standards Institute (CLSI, 2018). Sterile glass rods were used to streak the entire surface of Mueller–Hinton agar plates and antibiotic discs were applied aseptically on the Mueller–Hinton and incubated for 18 hours at 37°C using an antibiotic dispenser (Mast Diagnostics, UK). The antibiotic discs were used to determine the resistance patterns of the isolates against 11 selected antibiotics (**Table 1**). Bacterial isolates were classified as resistant (R), intermediate (I), or susceptible (S) by measurement of inhibition zone diameters as per the criteria of CLSI, 2021. The tested isolates were classified as MDR and XDR as described by Magiorakos et al. (2012).

**Table 1:** List of antibiotics used to determine the antibiotic resistance patterns of the bacterial isolates.

Categories		Class of antibiotics	Sub-classes of antibiotics	Antibiotics	
Cell wall inhibiting and disrupting membrane antibiotics	β-lactams	Penicillins	Natural penicillins	Penicillin-G (PEN, 10 µg)
			Aminopenicillins	Ampicillin/Cloxacillin (AX, 10 µg)
		Cephalosporins	Third generation	Ceftazidime (CAZ, 30 µg),
				Cefpodoxime (CPD, 10 µg),
			Fourth generation	Cefepime (CPM, 30 µg),
		Carbapenems		Meropenem (MRP, 10 µg),
	Glycopeptides			Vancomycin (VAN, 30 µg)
Nucleic acids inhibiting antibiotics	Inhibiting RNA synthesis antibiotics	Rifamycins		Rifampicin (RIF, 5 µg)
	DNA inhibitors antibiotics	Nitrofurans		Nitrofurantoin (NIT, 300 µg)
Protein synthesis inhibiting antibiotics	30S sub-unit inhibitors	Aminoglycosides		Streptomycin (STR, 10 µg)
		Phenolic derivatives		Chloramphenicol (CHL, 50 µg)

### Sequencing of 16S rRNA gene in confirmed MDR bacteria isolates

Sequencing of the 16S rRNA gene of the MDR bacterial isolates was performed and the isolates identified by BLASTn and phylogenetic analysis. Bacterial DNA was extracted using a DNA extraction kit (Qiagen, Hilden, Germany), as per manufacturer’s instructions. The extracted DNA was used as a template for PCR amplification of the 16S rRNA gene using 27F 5′-GAGTTTGATCCTGGCTCA-3′ and 1492R 5′-TACGGCTACCTTGTTACGACTT-3′ oligonucleotide primers (Fontana et al., 2005). A total of 50 μl reaction mixture was prepared with 2 μl of template DNA (100 ng/μl), 0.5μl each of forward and reverse primer pair, 25 μl of GoTaq Green Master Mix (Promega, Madison, United States), and nuclease-free water up to 50 μl. The PCR products were resolved by gel electrophoresis on 1.5% (w/v) agarose gel (Qiagen, Hombrechtikon, Switzerland). The gels were viewed under a gel imager (Bio-Rad Gel Doc XR System, United States). A QIAquick kit (Qiagen, Hilden, Germany) was used to purify amplicons according to the manufacturer’s instructions, and they were sequenced using the Sanger sequencing method (Macrogen, Netherlands). The nucleotide sequences were edited, assembled, and compared with those in the GenBank using BLASTn searches (Altschul et al., 1997) for identification of the bacteria. A phylogenetic tree was constructed with closely related GenBank sequences using the Bayesian inference method (MrBayes software v3.2.7; https://nbisweden.github.io/MrBayes/).

### Molecular detection of the inherited antimicrobial resistance genes

Genomic DNA of MDR bacterial isolates were used for PCR amplification of the antibiotic resistance genes. The PCR reaction mixture consisted of 12.5 μl GoTaq Green Master Mix (Promega, United States), 1 μl DNA extract (50 ng/µl), and 0.5 μl each of the forward and reverse primer pair, and was topped up with nuclease-free water to 25 μl. PCR cycling conditions and primer sequences are shown in **Table 2**. The reaction was done using a ProFlex PCR system (Applied Biosystems™, United States). The primers used were β-lactamase-encoding genes (*bla*TEM-1 and *bla*CMY-2), tetracycline-resistant genes (*tet*A and *tet*C), sulfonamide-resistant genes (*sul2*), trimethoprim-resistant genes (*dfrA7*), and aminoglycoside-resistant genes (*strA* and *aadA*) for antibiotics (**Supplementary Table S1**). The PCR products were resolved by electrophoresis on a 1.5% (w/v) agarose gel with 1× Tris-HCl-EDTA (TE) buffer and then allowed to run for 1 hour at 100 V. The gels were viewed under a gel imager (Bio-Rad Gel Doc XR System, United States) and photographed.

**Table 2:** Bacterial pathogen prevalence among fresh *O. niloticus* fish samples from different markets of five sub-counties in Nairobi.

Sub-county	Collected samples, N	Positive for bacterial pathogens, N (%)				
		*Proteus* spp.	*S. aureus*	*P. aeruginosa*	*V. cholerae*	*V. parahaemolyticus*
Kasarani	14	7 (50)	3 (21.43)	0 (0)	1 (7.14)	1 (7.14)
Makadara	14	6 (42.86)	0 (0)	0 (0)	0 (0)	0 (0)
Westlands	14	6 (42.86)	4 (28.57)	0 (0)	1 (7.14)	1 (7.14)
Embakasi	13	5 (38.46)	0 (0)	3 (23.08)	0 (0)	0 (0)
Lang’ata	13	6 (46.15)	0 (0)	0 (0)	0 (0)	0 (0)
Total	68	30 (44.12)	7 (10.29)	3 (4.41)	2 (2.94)	2 (2.94)

### Multiple antibiotic resistance among isolated bacteria

The multiple antibiotic resistance genotypes (MARGs) patterns of established pathogens with multiple resistance genes were analyzed as described by Titilawo et al. (2015). Multiple antibiotic-resistant phenotypes (MARPs) for each sampling site were generated for isolates that showed resistance to more than three classes of antibiotics following the method described by Wose et al. (2010).

### Statistical analysis

Statistical analysis was performed using Minitab v17.1 statistical software (Minitab, LLC). The chi-squared test was used to test differences. Significance tests were considered statistically significant when *p* values were < 0.05. Correlation coefficient was performed using Microsoft Excel 2016 (Microsoft Corporation).

## Results

### Prevalence of bacterial isolates in fresh Nile tilapia samples from retail markets

The prevalence and contamination rates of *Proteus* spp. ranged from 38.46% to 50% and *S. aureus* ranged from 0% to 28.57% for the five different sub-counties under study. *P. aeruginosa* was detected in fish samples from only Embakasi sub-county, with a prevalence of 23.08%. With regards to *V. cholerae* and *V. parahaemolyticus*, prevalence of 7.14% was obtained from fish samples collected from two sub-counties, Kasarani and Westlands (**Table 3**). Overall, *Proteus* spp. (44.12%) was the most prevalent bacterial pathogen followed by *S. aureus* (28.57%) and *P. aeruginosa* (23.08%). The least prevalent bacterial pathogens were *V. cholerae* and *V. parahaemolyticus* (7.14%) (**Table 2**).

**Table 3:** Phenotypic resistance pattern of *Proteus* spp., *S. aureus*, *P. aeruginosa*, *V. cholerae*, and *V. parahaemolyticus* to 11 antimicrobial agents.

Antimicrobial class	Antibiotics	Bacteria isolates														
		*Proteus* spp. (n = 30)			*S. aureus* (n = 7)			*P. aeruginosa* (n = 3)			*V. cholerae* (n = 2)			*V. parahaemolyticus* (n = 2)		
		R	I	S	R	I	S	R	I	S	R	I	S	R	I	S
Penicillin	PEN	33.3	40	26.7	57.1	42.9	0	100	0	0	100	0	0	100	0	0
Glycopeptides	VAN	70	6.7	23.3	71.4	28.6	0	100	0	0	100	0	0	100	0	0
Rifamycins	RIF	73.3	16.7	10	100	0	0	66.7	33.3	0	0	100	0	50	50	0
β-lactam	AX	70	26.7	3.3	100	0	0	66.7	33.3	0	100	0	0	100	0	0
Cephalosporin (fourth)	CPM	0	6.7	93.3	0	28.6	71.4	0	66.7	33.3	0	0	100	0	0	100
Cephalosporin (third)	CPD	50	20	30	85.7	14.3	0	33.3	66.7	0	100	0	0	0	100	0
Phenicol	CHL	10	10	80	0	14.3	85.7	0	0	100	50	50	0	0	0	100
Nitrofuran	NIT	6.7	33.3	60	14.3	14.3	71.4	0	33.3	66.7	50	50	0	0	0	100
Cephalosporin (third)	CAZ	13.3	16.7	70	71.4	28.6	0	100	0	0	0	100	0	0	0	100
Carbapenems	MRP	36.7	0	63.3	100	0	0	100	0	0	0	0	100	50	0	50
Aminoglycosides	STR	60	20	20	28.6	42.9	28.6	100	0	0	100	0	0	50	50	0
	Mean	38.5	17.9	43.6	57.1	19.5	23.4	60.6	21.2	18.2	54.5	27.3	18.2	40.9	18.2	40.9

PEN, penicillin-G (10 µg); VAN = vancomycin (30 µg); RIF, rifampicin (5 µg); AX, ampicillin/cloxacillin (10 µg); CPM, cefepime (30 µg); CPD, cefpodoxime (10 µg); CHL, chloramphenicol (50 µg); NIT, nitrofurantoin (300 µg); CAZ, ceftazidime (30 µg); MRP, meropenem (10 µg); STR, streptomycin (10 µg); R, resistant; I, intermediate; S, sensitive.

### Antibiotic susceptibility testing according to the species of isolated bacteria

The distribution of antimicrobial resistance pattern of *Proteus* spp., *S. aureus*, *P. aeruginosa*, *V. cholerae* and *V. parahaemolyticus* is presented in **Table 3**. None of the bacterial isolates from the 5 different bacterial pathogens were resistant to cefepime (fourth-generation cephalosporin). The antibiotic resistance profile among the *Proteus* species isolates demonstrated varying levels of resistance against the 11 antibiotics. The highest level of resistance was observed for rifampicin (73.3%) followed by vancomycin and ampicillin/cloxacillin (70%), streptomycin (60%), and cefpodoxime (50%). With regard to S. aureus, the antibiotics with the highest resistance rates were rifampicin, ampicillin/cloxacillin, and meropenem (100%) followed by cefpodoxime (85.7%), vancomycin and ceftazidime (71.4%), and penicillin-G (57.1%). In *P. aeruginosa*, all the isolates were resistant to penicillin-G and vancomycin (100%) followed by ceftazidime, meropenem, and streptomycin (66.7%), and cefpodoxime (33.3%), but were not resistant to any of the remaining antibiotics (cefepime, chloramphenicol, and nitrofurantoin). For *V. cholerae*, 100% were resistant to penicillin-G, vancomycin, ampicillin/cloxacillin, cefpodoxime, and streptomycin. For *V. parahaemolyticus*, 100% were resistant to penicillin-G, vancomycin, and ampicillin/cloxacillin. Intermediate percentages ranged from 6.7% to 100% (**Table 3**). No resistance to chloramphenicol was noted for any of the bacterial pathogens except *Proteus* species isolates.

### Multidrug-resistant patterns of bacterial isolates

The MDR, XDR, and MAR index distribution of bacterial isolates is presented in **Table 4**. Multidrug resistance was present among all five bacterial pathogens (**Table 4**). As revealed by the antibiogram typing, *Proteus* spp. (56.7%), *S. aureus* (71.4%), *P. aeruginosa* (33.3%), *V. cholerae* (100%) and *V. parahaemolyticus* (100%) were multidrug resistant. XDR was expressed only in *Proteus* spp. (23.3%), *S. aureus* (28.6%), and *P. aeruginosa* (66.7%). No PDR was expressed by the tested bacterial isolates. Among the antibiogram types, PEN-VAN-RIF-AX-CPD-MRP-STR showed the highest prevalence (23.3%, seven isolates) in *Proteus* spp., with MAR of 0.64. A total of 6.7% of *Proteus* spp. were resistant to three antibiotics (VAN, RIF, AX) which belonged to three different groups of antimicrobials with a MAR index of 0.27. A total of 1/7 (14.3%) *S. aureus* were resistant to four antibiotics (RIF, AX, CPD, MRP) which belonged to four different groups of antimicrobials with a MAR index of 0.36. Furthermore, 2/7 (28.6%) *S. aureus* were resistant to seven antibiotics (PEN, VAN, RIF, AX, CPD, CAZ, MRP) which belonged to six different groups of antimicrobials with a MAR index of 0.64. A total of 1/3 (33.3%) *P. aeruginosa* were resistant to six antibiotics (PEN, VAN, CPD, CAZ, MRP, STR) which belonged to five different groups of antimicrobials with a MAR index of 0.55. Furthermore, 2/3 (66.7%) of *P. aeruginosa* were resistant to seven antibiotics (PEN, VAN, RIF, AX, CAZ, MRP, STR) which belonged to seven different groups of antimicrobials with a MAR index of 0.64. V*. cholerae* showed a different MDR pattern (PEN, VAN, AX, CPD, CHL, STR and PEN, VAN, AX, CPD, NIT, STR) but with similar MAR index (0.55). A total of 1/2 (50%) *V. parahaemolyticus* were resistant to four antibiotics (PEN, VAN, RIF, AX) which belonged to four different groups of antimicrobials with a MAR index of 0.36. Furthermore, 1/2 (50%) *V. parahaemolyticus* were resistant to five antibiotics (PEN, VAN, AX, MRP, STR) which belonged to four different groups of antimicrobials with a MAR index of 0.45 (**Table 4**). Overall, diverse patterns of resistance to different classes of antibiotics were observed among *Proteus* spp., *S. aureus*, *P. aeruginosa*, *V. cholerae*, and *V. parahaemolyticus* isolates (**Table 4**).

**Table 4:** Distribution of multiple antibiotic resistances in *Proteus* spp., *S. aureus*, *P. aeruginosa*, *V. cholerae*, and *V. parahaemolyticus*.

Microorganism	Number of antimicrobial class	Number of antibiotics	Resistance phenotypes	MDR prevalence (%)	XDR Prevalence (%)	MAR index
*Proteus* spp. (n = 30)						
	3	3	VAN^R^, RIF^R^, AX^R^	2 (6.7)	–	0.27
	4	4	VAN^R^, AX^R^, CHL^R^, STR^R^	2 (6.7)	–	0.36
	4	4	RIF^R^, AX^R^, CHL^R^, STR^R^	1 (3.3)	–	0.36
	4	4	PEN^R^, VAN^R^, RIF^R^, CPD^R^	1 (3.3)	–	0.36
	4	4	VAN^R^, RIF^R^, AX^R^, CPD^R^	1 (3.3)	–	0.36
	4	4	RIF^R^, CPD^R^, NIT^R^, STR^R^	1 (3.3)	–	0.36
	4	4	RIF^R^, AX^R^, MRP^R^, STR^R^	1 (3.3)	–	0.36
	5	5	VAN^R^, RIF^R^, CPD^R^, NIT^R^, STR^R^	1 (3.3)	–	0.45
	5	5	VAN^R^, RIF^R^, AX^R^, MRP^R^, STR^R^	3 (10)	–	0.45
	4	5	VAN^R^, RIF^R^, AX^R^, CPD^R^, CAZ^R^,	2 (6.7)	–	0.45
	7	7	PEN^R^, VAN^R^, RIF^R^, AX^R^, CPD^R^, MRP^R^, STR^R^		7 (23.3)	0.64
	6	7	PEN^R^, VAN^R^, RIF^R^, AX^R^, CPD^R^, CAZ^R^, STR^R^	2 (6.7)		0.64
				17 (56.7)	7 (23.3)	
S. aureus (n = 7)						
	4	4	RIF^R^, AX^R^, CPD^R^, MRP^R^	1 (14.3)		0.36
	5	6	PEN^R^, RIF^R^, AX^R^, CPD^R^, CAZ^R^, MRP^R^	1 (14.3)		0.55
	5	6	VAN^R^, RIF^R^, AX^R^, CPD^R^, CAZ^R^, MRP^R^	1 (14.3)		0.55
	7	7	PEN^R^, VAN^R^, RIF^R^, AX^R^, CPD^R^, MRP^R^, STR^R^		1 (14.3)	0.64
	7	7	VAN^R^, RIF^R^, AX^R^, NIT^R^, CAZ^R^, MRP^R^, STR^R^		1 (14.3)	0.64
	6	7	PEN^R^, VAN^R^, RIF^R^, AX^R^, CPD^R^, CAZ^R^, MRP^R^	2 (28.6)		0.64
				5 (71.4)	2 (28.6)	
P. aeruginosa (n = 3)						
	5	6	PEN^R^, VAN^R^, CPD^R^, CAZ^R^, MRP^R^, STR^R^	1 (33.3)		0.55
	7	7	PEN^R^, VAN^R^, RIF^R^, AX^R^, CAZ^R^, MRP^R^, STR^R^		2 (66.7)	0.64
				1 (33.3)	2 (66.7)	
V. cholerae (n = 2)						
	6	6	PEN^R^, VAN^R^, AX^R^, CPD^R^, CHL^R^, STR^R^	1 (50)		0.55
	6	6	PEN^R^, VAN^R^, AX^R^, CPD^R^, NIT^R^, STR^R^	1 (50)		0.55
				2 (100)		
V. parahaemolyticus (n = 2)						
	4	4	PEN^R^, VAN^R^, RIF^R^, AX^R^	1 (50)		0.36
	4	5	PEN^R^, VAN^R^, AX^R^, MRP^R^, STR^R^	1 (50)		0.45
				2 (100)		

PEN, penicillin-G (10 µg); VAN, vancomycin (30 µg); RIF, rifampicin (5 µg); AX, ampicillin/cloxacillin (10 µg); CPM, cefepime (30 µg); CPD, cefpodoxime (10 µg); CHL, chloramphenicol (50 µg); NIT, nitrofurantoin (300 µg); CAZ, ceftazidime (30 µg); MRP, meropenem (10 µg); STR, streptomycin (10 µg). MDR, multidrug resistance; XDR, extensive drug resistance;

MAR, multiple antibiotic resistance index. MDR represents non-susceptible to ≥1 agent in ≥3 antimicrobial categories, while XDR represents non-susceptible to ≥1 agent in all but ≤2 categories.

### Identification of MDR bacteria using BLASTn analysis

The sequences of 16S rRNA genes of MDR isolates of bacterial pathogens were compared with strains in the GenBank to determine the degree of similarity between them and closely related genotypes. BLASTn results revealed the percentage of similarity between MDR isolates and closely related bacteria in the GenBank (**Supplementary Table S2**). The 16S rRNA 1465 bp amplicon sequences were registered in GenBank and the accession numbers are provided in **Supplementary Table S2**. From the 16S rRNA sequences, BLASTn analysis showed the four MDR *Vibrio* isolates belonged to *V. cholerae* (accession numbers OP293360.1 and OP293361.1) and *V. parahaemolyticus* (accession numbers OP293358.1 and OP293359.1). Using BLASTn analysis and the globally published NCBI database, three MDR isolates of *Pseudomonas* were confirmed as *P. aeruginosa*, seven MDR isolates of *Staphylococcus* were confirmed as *S. aureus*, and three Proteus spp. were confirmed as *P. mirabilis* and other *Proteus* spp. (**Supplementary Table S2**). The accession numbers of the MDR and XDR bacterial isolates for the current study are presented in **Supplementary Table S2**.

### Phylogenetic analysis

Phylogenetic analysis of 16S rRNA sequences of the MDR bacterial isolates showed distinct clustering and each phylogenetic tree had the same respective nodes showing that they evolved from the same ancestor.

### *Proteus* species

**Figure 1:**
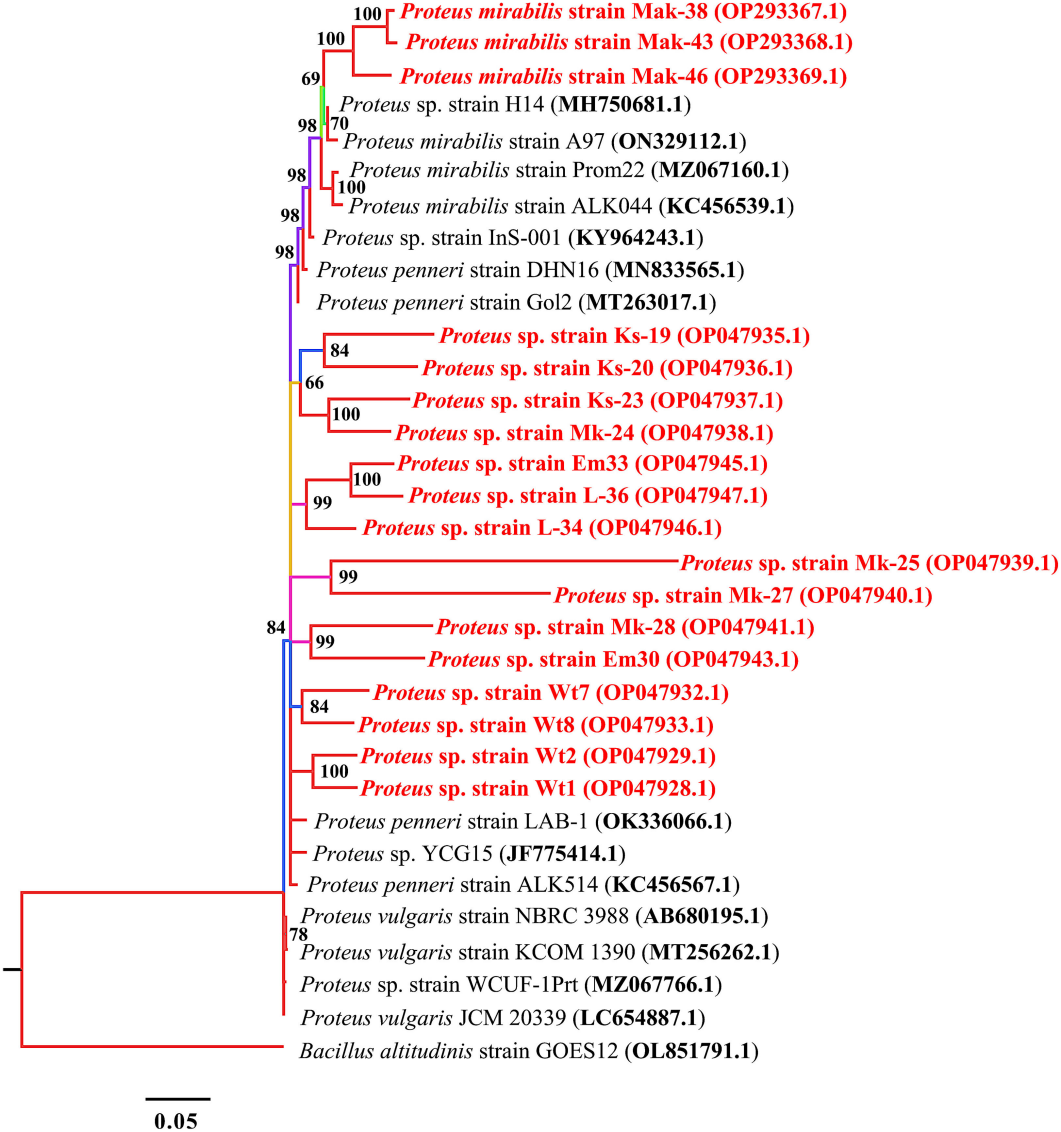
Phylogenetic tree built by MrBayes v3.2.7 using 14 16S rRNA sequences of the genus *Proteus*. New isolates *P. mirabilis* strains, Mak-38, Emb-43, and Lan-46, and *Proteus* spp. strains Ks-19, Ks-20, Ks-23, Mk-24, Mk-25, Mk-27, Mk-28, Em30, Em33, L-34, L-36, Wt1, Wt2, Wt7, and Wt8 are shown in red. Numbers indicated on the nodes are percent posterior probabilities showing statistical support for each node. Branches are colored based on percent posterior probabilities. The scale bar below the tree indicates the number of expected changes (or substitutions) per site. The *Bacillus altitudinis* strain GOES12 (OL851791.1) was used as an outgroup in the phylogenetic tree.

Based on 16S rRNA sequencing and subsequent BLAST analysis, all strains shared more than 96% sequence homology with different types of strains of the genus *Proteus*. Out of the 18 *Proteus* spp. isolates, three (accession numbers OP293367.1, OP293368.1, and OP293369.1) shared more than 98% similarity with *Proteus mirabilis* 16S rRNA gene sequences obtained on the NCBI (**Figure 1**). Other *Proteus* spp. (accession numbers OP047928.1 and OP047929.1) shared 84% similarity with *Proteus penneri*. The isolates EM33 (OP047945.1) and L-36 (OP047947.1) had similar sequence identity although the isolates were obtained from different locations.

#### Staphylococcus aureus

**Figure 2:**
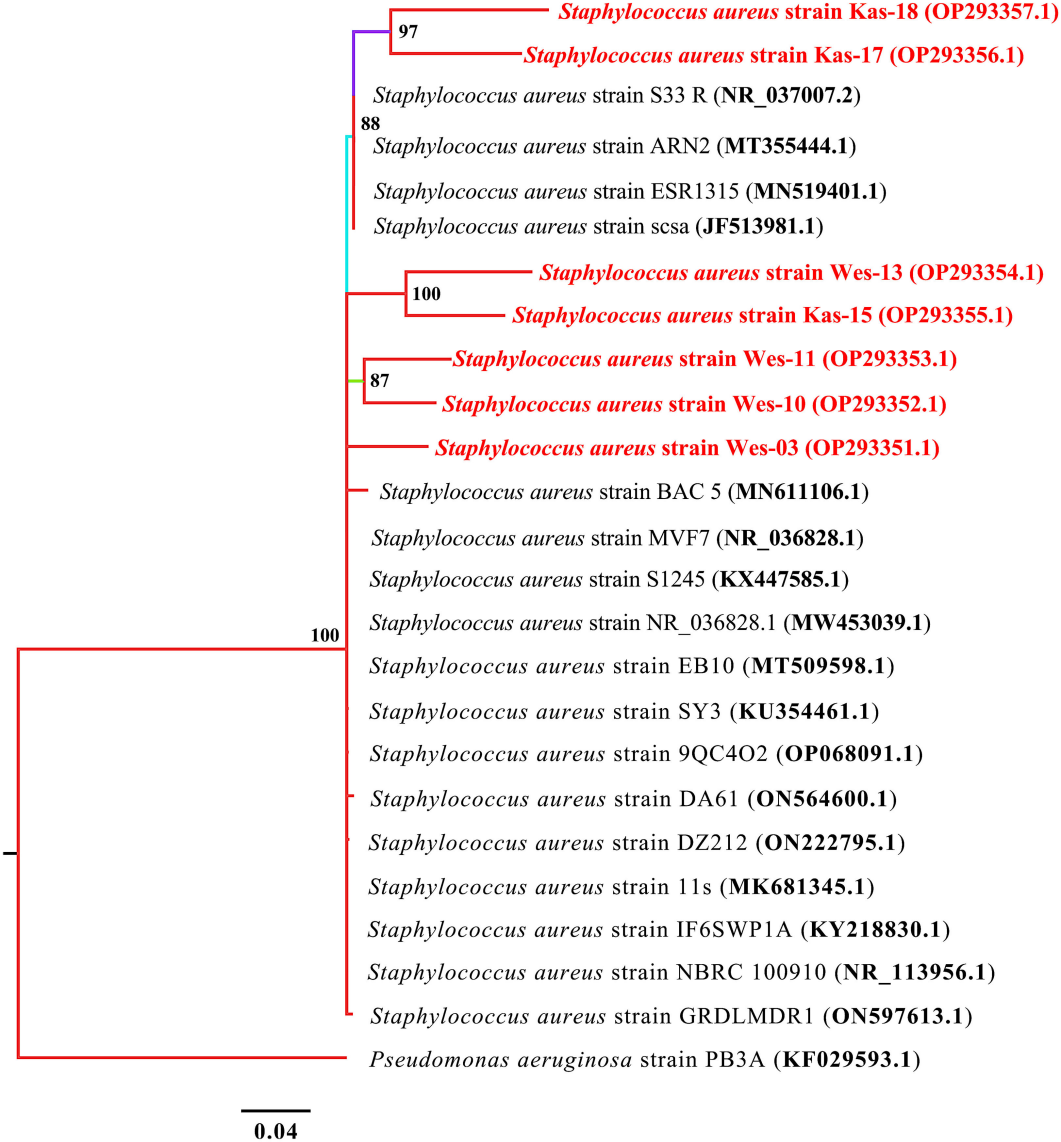
Phylogenetic tree built by MrBayes v3.2.7 using 17 16S rRNA sequences of *S. aureus* species. New isolates *S. aureus* strains Wes-03, Wes-10, Wes-11, Wes-13, Kas-15, Kas-17, and Kas-18 are shown in red. Numbers indicated on the nodes are percent posterior probabilities showing statistical support for each node. Branches are colored based on percent posterior probabilities. The scale bar below the tree indicates the number of expected changes (or substitutions) per site. The *P. aeruginosa* strain PB3A (KF029593.1) was used as an outgroup in the phylogenetic tree.

In the neighbour-joining phylogenetic trees based on 16S rRNA gene sequences, the MDR isolates formed clades with the related strains from the database. All seven isolates shared nodes, with bootstrap values ranging from 87% to 100% (**Figure 2**). All seven isolates were closely related to *S. aureus* strains in the databases and thus were identified as *S. aureus*.

#### Pseudomonas aeruginosa

**Figure 3:**
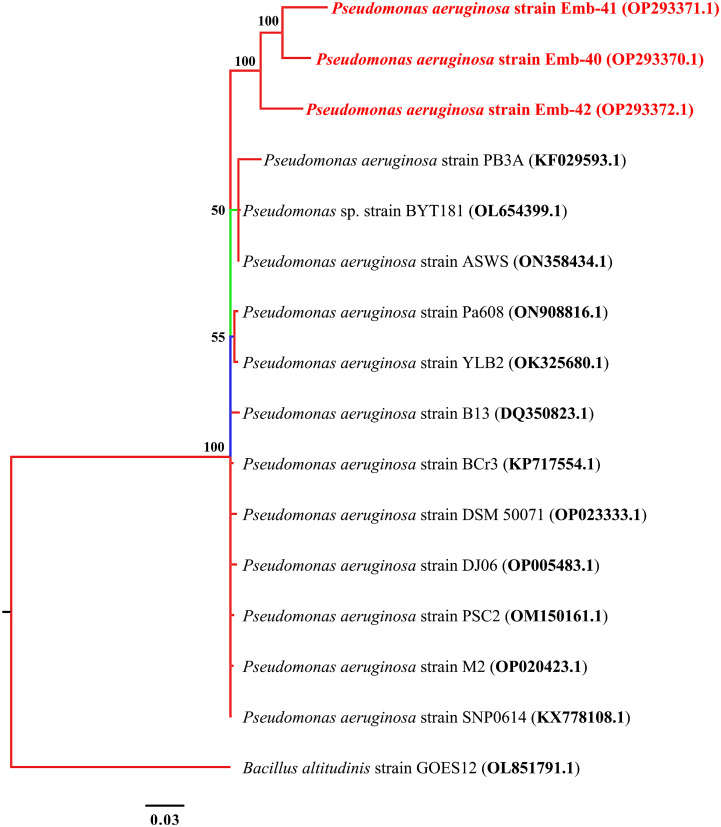
Phylogenetic tree built by MrBayes v3.2.7 using 21 16S rRNA sequences of *P. aeruginosa* species. New isolates *P. aeruginosa* strains Emb-40, Emb-41, and Emb-42 are shown in red. Numbers indicated on the nodes are percent posterior probabilities showing statistical support for each node. Branches are colored based on percent posterior probabilities. The scale bar below the tree indicates the number of expected changes (or substitutions) per site.

Based on the phylogenetic analysis using 16S rRNA, the three MDR isolates of *P. aeruginosa* exhibited a high degree of similarity with one another, with similarity percentages between 98.5% and 100%. *P. aeruginosa* strains Emb-40 and Emb-41 had similar identity although they were obtained from different markets at the same location (**Figure 3**).

#### *Vibrio* species

**Figure 4:**
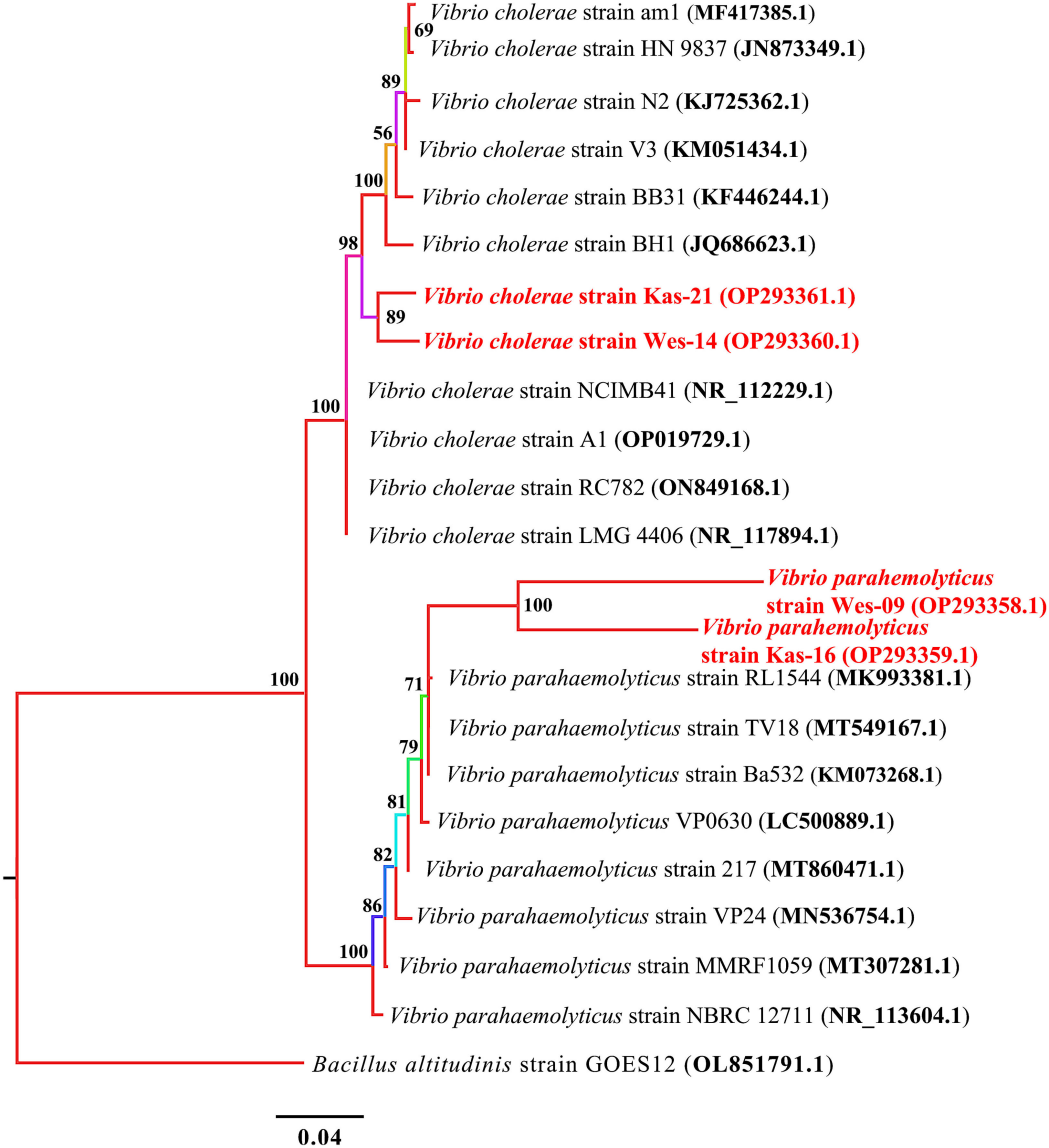
Phylogenetic treebuilt by MrBayes v3.2.7using 10 and 8 16S rRNA sequences of *V. cholerae* and *V. parahaemolyticus*, respectively. New isolates *V. cholerae* strains Wes-14 and Kas-21, and *V. parahaemolyticus* strains Wes-06 and Kas-16 are shown in red. Numbers indicated on the nodes are percent posterior probabilities showing statistical support for each node. Branches are colored based on percent posterior probabilities. The scale bar below the tree indicates the number of expected changes (or substitutions) per site. The *B altitudinis* strain GOES12 (OL851791.1) was used as an outgroup in the phylogenetic tree.

A phylogenetic tree of 16S rRNA sequences showed that all the tested samples were grouped into two main clusters. Each of the two species identified, *V. cholerae* and *V. parahaemolyticus*, clustered separately (**Figure 4**).

#### Detection of antimicrobial resistance genes by PCR

**Figure 5:**
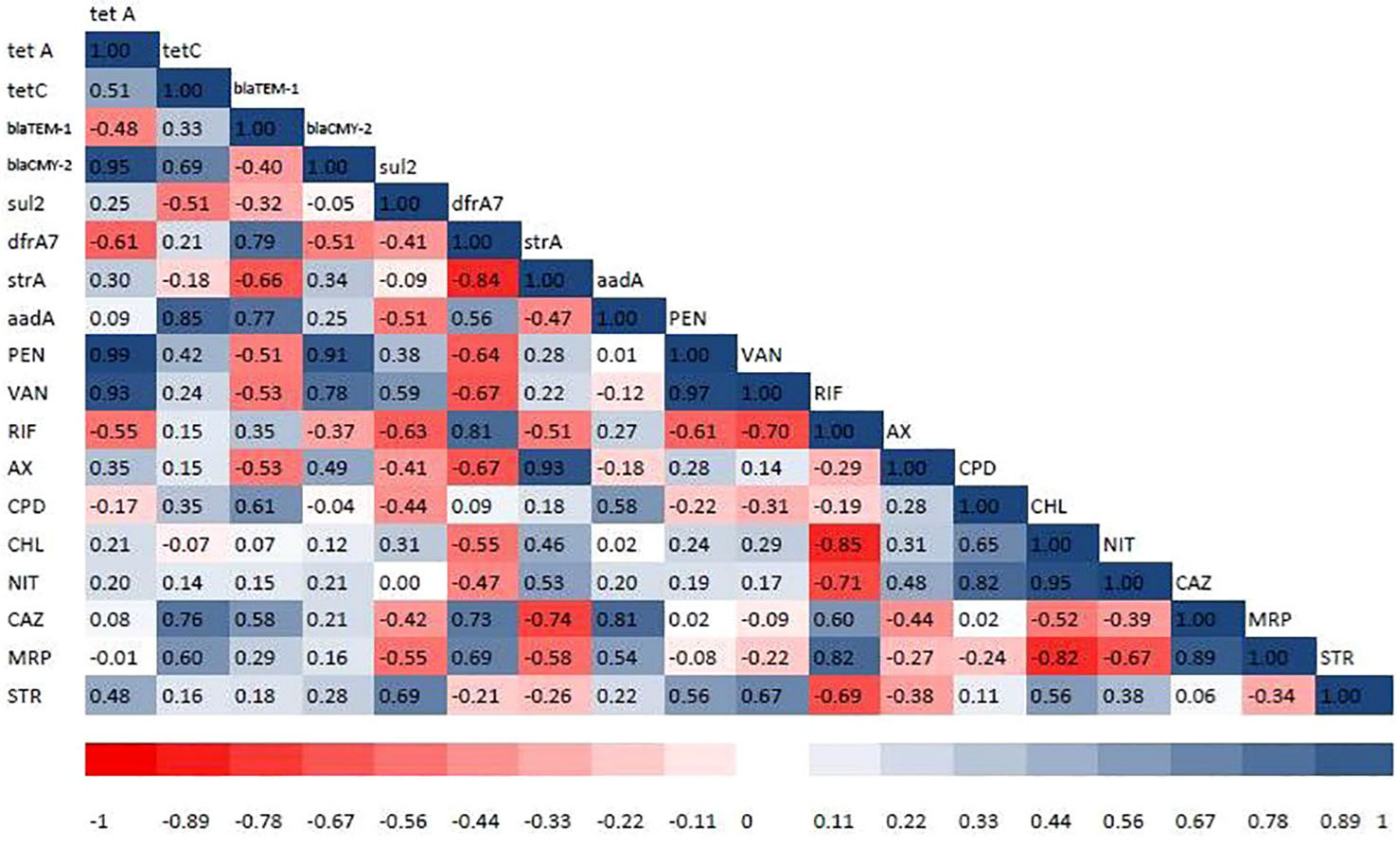
Heat map showing correlation coefficient (r) among the tested antibiotics and antibiotic resistance genes detected in bacterial isolates. The intensity of colors indicates the numerical value of the correlation coefficient (r). Red and blue colors refer to the negative and positive correlations, respectively. PEN, penicillin-G (10 µg); VAN, vancomycin (30 µg); RIF, rifampicin (5 µg); AX, ampicillin/cloxacillin (10 µg); CPM, cefepime (30 µg); CPD, cefpodoxime (10 µg); CHL, chloramphenicol (50 µg); NIT, nitrofurantoin (300 µg); CAZ, ceftazidime (30 µg); MRP, meropenem (10 µg); STR, streptomycin (10 µg). *tet*A and *tet*C = tetracycline-resistant genes, *bla*TEM-1 and *bla*CMY-2 = β-lactamases-encoding genes, *sul*2 = sulphonamide-resistant gene, *dfr*A7 = trimethoprim-resistant gene, *str*A = streptomycin-resistant gene, and *aad*A = aminoglycoside-resistant genes.

PCR was used to determine the drug resistance genes of MDR isolates of the different bacterial pathogens. The distribution of antimicrobial-resistant genes among multidrug-resistant (MDR) *Proteus* spp.*, S. aureus*, *P. aeruginosa*, *V. cholerae*, and *V. parahaemolyticus* are presented in **Table 5**. All the MDR isolates of the different bacteria species tested positive for *bla*TEM-1, *bla*CMY-2, *Sul*2, *str*A, *aad*A, *tet*A, *tet*C, and *dfr*A7. However, *sul*2 gene was not amplified in two *S. aureus* isolates (accession numbers OP293352.1 and OP293356.1). **Supplementary Figure S1** shows a representative agarose gel of the amplification of *Proteus* spp., *S. aureus*, *P. aeruginosa*, *V. cholerae* and *V. parahaemolyticus* tested antibiotic-resistant genes. The correlation coefficient (r) was assessed between phenotypic and genotypic multidrug resistance in *bacterial* isolates. The results revealed significant positive correlations between: blaCMY-2 gene and *tet*A (r = 0.95), AX and *str*A gene (r = 0.93), PEN and blaCMY-2 gene (r = 0.91), aad*A gene and* tet*C* (r = 0.85), RIF and *dfr*A7 (r = 0.81), CAZ and aad*A gene (*r = 0.81*), VAN* and blaCMY-2 gene (r = 0.78), and *CAZ* and *dfr*A7 (r = 0.73) (**Figure 5**).

**Table 5:** Distribution of antimicrobial-resistant genes in MDR isolates of the different bacteria pathogens.

MDR isolates	Antibiotic-resistant associated genes (%)							
	*tet*A	*tet*C	*bla*TEM-1	*bla*CMY-2	*sul*2	*dfr*A7	*str*A	*aad*A
*Proteus* spp. (n = 22)	24 (100)	24 (100)	24 (100)	24 (100)	24 (100)	24 (100)	24 (100)	24 (100)
*S. aureus* (n = 7)	7 (100)	7 (100)	7 (100)	7 (100)	5 (71.4)	7 (100)	7 (100)	7 (100)
*P. aeruginosa* (n = 3)	3 (100)	3 (100)	3 (100)	3 (100)	3 (100)	3 (100)	3(100)	3 (100)
*V. cholerae* (n = 2)	2 (100)	2 (100)	2 (100)	2 (100)	2 (100)	2 (100)	2 (100)	2 (100)
*V. parahaemolyticus* (n = 2)	2 (100)	2 (100)	2 (100)	2 (100)	2 (100)	2 (100)	2 (100)	2 (100)
Total (n = 36)	36 (100)	36 (100)	36 (100)	36 (100)	36 (94.4)	36 (100)	36 (100)	36 (100)

tetA and tetC, tetracycline-resistant genes; blaTEM-1 and blaCMY-2, β-lactamases-encoding genes; sul2, sulphonamide-resistant gene; dfrA7, trimethoprim-resistant gene; strA, streptomycin-resistant gene; aadA, aminoglycoside-resistant gene.

## Discussion

In this study the prevalence of contamination of fresh *O. niloticus* fish by the selected foodborne pathogenic bacteria was investigated. In addition, the antibiotic resistance pattern of the isolates, the presence of resistant genes and the genetic diversity of the identified MDR resistant isolates of the pathogens was determined. The current study revealed the presence of *Proteus* spp., *S. aureus*, *P. aeruginosa*, *V. cholerae*, and *V. parahaemolyticus* in fresh *O. niloticus* fish samples obtained from retail markets in Nairobi, Kenya. The presence of the bacterial contaminants is a reflection of the wide range of infections to which consumers of *O. niloticus* fish are exposed to, especially if the fish is undercooked prior to consumption. The presence in fish of some of these bacteria, such as *Proteus* spp. and *S. aureus*, have been reported to cause food poisoning outbreaks. The prevalence of *Proteus* spp. and *S. aureus* in the present study was 44.12% and 10.29%, respectively, which is within the range reported by previous studies from different countries (Kusunoki et al., 1998; Omoe et al., 2002; Saito et al., 2011; Bujjamma and Padmavathi, 2015). The current prevalence of *Proteus* spp. and *S. aureus* represents a major public health and economic burden for the country. The source of *Proteus* spp. and *S. aureus* in *O. niloticus* fish could be human contamination, as they are not part of the known bacterial flora of fish. The presence of *Proteus* spp. and *S. aureus* from *O. niloticus* in retail markets is due to unhygienic handling during processing, transportation, and storage (Titilawo et al., 2015). Thus, it is necessary for regulatory agencies to increase the robustness with which they monitor and enforce the microbial safety of fish and other fish products, and for vendors to strictly adhere to proper handling practices. It is also necessary to sensitize consumers to the need to ensure that they cook their fish well to allow for the removal of bacterial contaminants before consumption.

*P. aeruginosa* is naturally found in aquatic environments. In the current study, the prevalence of *P. aeruginosa* (6.8%) was higher than in other studies reported for *O. niloticus* (5.1%) in Uganda (Wamala et al., 2018), and for salmon (1.1%), shrimp (0.9%), and *O. niloticus* (2.3%) (Tate et al., 2022). Marijani (2022) reported *Pseudomona*s spp. (1%) in marine and freshwater fish in Tanzania. *Vibrio* spp. are found in fish and fish environments and may be harmful to both wild and cultured fish (Gauthier, 2015). The findings of this study showed that *Vibrio* were the least prevalent, at 4.5%, which is lower than in studies by Wamala et al. (2018), where *V. cholerae* (12.8%) was reported in O. niloticus; *V. parahaemolyticus* (11.22%) in ready-to-eat foods, shrimp and fish in China (Xie et al., 2020). The presence of Vibrio in aquatic environments indicates contamination by human waste. *V. parahaemolyticus* is associated with human vibriosis and occurs mainly due to the ingestion of undercooked fish or fish products (Iwamoto et al., 2010; Callol et al., 2015).

The rates of resistance of the various classes of antimicrobials agents used among the bacterial pathogens recovered in this study ranged from 0%–100%, with high-level resistance to ampicillin/cloxacillin, vancomycin and streptomycin. The high-level resistance to ampicillin, a derivative of penicillin, could be due to easy access to penicillin and its frequent use in aquaculture. Such resistant phenotypes indicate antibiotic failure, should these members of antibiotics be used in the treatment of any disease implicated by any of the characterized members of organisms. All bacterial isolates were sensitive to cefepime, which is a fourth-generation cephalosporin. Cefepime has higher activity against both Gram-positive and Gram-negative bacteria than third-generation cephalosporins and, because it is new to the market, it is possible that there is zero to low use in aquaculture farming in Kenya.

Gram-negative bacteria use antibacterial resistance mechanisms such as drug efflux, inactivating drugs, limiting uptake of drugs, and modifying the drug target, unlike Gram-positive bacteria, which lack lipopolysaccharide in the outer membrane and hence cannot pump out drugs from their cell wall (Chancey et al., 2012; Mahon et al., 2014). The current study revealed that *S. aureus* was resistant to vancomycin, which is in agreement with a study by Reygaert (2018); however, the mechanism is yet to be explained. Previous studies indicate that the bacteria produce a thickened cell wall; hence, the drug cannot enter the cell and as a result it provides an intermediate resistance to vancomycin (Lambert, 2002; Miller et al., 2014). Formation of biofilm by pathogenic bacteria, e.g., *P. aeruginosa*, protects the bacteria from antimicrobial agents and the host immune system. Horizontal gene transfer is enabled by the proximity of the bacterial cells in biofilms and hence the transfer of antimicrobial resistance genes (Mah, 2012; Van Acker et al., 2014).

The prevalence of antibiotic resistance for *P. aeruginosa* was highest in penicillin (100%), vancomycin (100%), ceftazidime (100%), meropenem (100%), and streptomycin (100%). The reported resistance is due to restricted outer membrane permeability of the antimicrobials, efflux systems that pump antimicrobials out of the cell, and synthesis of antibiotic-inactivating factors such as β-lactamases. The resistance of *P. aeruginosa* to the β-lactam antibiotics, including penicillin (first, second, and third generations) and cephalosporin (such as cefotaxime), is mainly attributed to the extended spectrum β-lactamases (ESBLs). *bla*CTX-M and *bla*TEM are the main extended spectrum β-lactamase genes that induce such type of resistance (Peymani et al., 2017). In addition, *P. aeruginosa* are capable of producing carbapenemase enzymes, which makes them resistant (Breidenstein et al., 2011). Furthermore*, P. aeruginosa* produces phenazine compounds, which are biologically active substances involved in bacterial competitiveness and virulence in both human and animal hosts (Mavrodi et al., 2001). The outer membrane proteins (L-lipoproteins) of *P. aeruginosa* are associated with bacterial resistance to antiseptics and antibiotics (Nikbin et al., 2012).

The prevalence of antibiotic-resistant *V. parahaemolyticus* is an important concern for public human health and veterinary medicine (Zanetti et al., 2001; de Melo et al., 2011; Sudha et al., 2014). High level of resistance of *V. parahaemolyticus* isolates to penicillin (100%), vancomycin (100%), ampicillin/cloxacillin (100%), meropenem (50%), and streptomycin (50%) was reported in this study; however, the isolates were sensitive to third- and fourth-generation cephalosporins, such as cefepime, cefpodoxime, nitrofurantoin, and ceftazidime. These findings are similar to reports from Korea and Malaysia that revealed high levels of resistance of *V. parahaemolyticus* to penicillin, vancomycin, ampicillin/cloxacillin, meropenem, and streptomycin (Jun et al., 2012; Kang et al., 2017; Tan et al., 2017). *V. cholerae* also showed high resistance to penicillin (100%), vancomycin (100%), ampicillin/cloxacillin (100%), and streptomycin (100%), and this is similar to previous studies by Das et al. (2020). Penicillins, including ampicillin, are the most commonly used antibiotic agents in aquaculture and therefore our results suggest that penicillins should not be used for clinical treatment of *V. parahaemolyticus* infections, whereas third- and fourth-generation cephalosporins are still useful for treatment. Other types of treatment that would play a role in reducing the prevalence of *Vibrio* spp. include low-temperature treatment, use of saline, and ultrasound (Zhou et al., 2002). The use of sugar, lemon juice, citric acid, or vinegar has also been linked with decreased contamination with *Vibrio* spp. in fish and shellfish (Borazjani et al., 2003; Ibrahim et al., 2018). Marinating fish prior to consumption is an additional method that can be used to reduce contamination with *Vibrio* spp.

*S. aureus* is widespread in the environment and in the human body. *S. aureus* infections, especially the one caused by methicillin-resistant *S. aureus* strains (MRSA), are a threat to public health due to the emergence of multidrug-resistant strains (Kaplan et al., 2005). Most of the isolates in this study showed resistance to penicillin, vancomycin, rifampicin, cefpodoxime, ceftazidime, ampicillin/cloxacillin, and meropenem, whereas levels of resistance to nitrofurantoin and streptomycin were lower. Moreover, none of the isolates were resistant to cefepime and chloramphenicol. Currently, vancomycin is the drug of choice for the treatment of *S. aureus* infections. In the current study 71.4% of *S. aureus* isolates were resistant to vancomycin, and this could be an alert for the emergence of multidrug-resistant *S. aureus* infections, especially after the consumption of undercooked fish.

Multidrug-resistant foodborne bacteria pathogens are a major public health and economic concern worldwide. The presence of MDR bacteria in fresh *O. niloticus* fish in the current study is a cause for worry for several reasons. Undercooked fish could expose consumers to colonization by and infection with these MDR bacteria. This has the potential for dissemination of the resistance genes from MDR bacterial pathogens to the microbiota with which they co-colonize the gut (Duedu et al., 2017). It is also possible that the resistance traits could be transferred to other microorganisms in circulation when colonized persons shed them in fecal matter. MDR bacteria could also spread from fish markets to hospital environments through cockroaches and other insects, and other vehicles for transmission of foodborne pathogens (Tetteh-Quarcoo et al., 2013; Futagbi et al., 2017; Donkor, 2019; Obeng-Nkrumah et al., 2019). The risk of transmission could be pronounced in cases of close proximity between markets and healthcare facilities. These pathogens could be disseminated further in the hospital facilities and negatively impact disease outcomes of patients and healthcare costs (Tetteh-Quarcoo et al., 2013; Obeng-Nkrumah et al., 2015; Tette et al., 2016; Donkor et al., 2018; Donkor and Kotey, 2020). Continued research on the antibiotic resistance of pathogenic bacteria that infect fish is needed because it is essential for controlling the occurrence of multidrug-resistant bacteria and for the selection of appropriate therapeutic agents. The high percentage of MDR *S. aureus* in the current study is slightly higher than in a previous study conducted in Egypt (Badawy et al., 2022) where 83.3% of *S. aureus* isolates were MDR.

This study was, to the best of our knowledge, the first to calculate and report the MAR index for *Proteus* spp., *S. aureus*, *P. aeruginosa*, *V. cholerae*, and *V. parahaemolyticus* isolated from fresh *O. niloticus* fish. The MAR index ranged from 0.27–0.64, indicating that all the isolates have repeatedly been exposed to antibiotics. All the bacteria pathogens tested in the present study showed a MAR index of more than 0.2, indicating a high risk of contamination that is potentially harmful to human health (Tanil et al., 2005). The dissemination of these resistant clones can pose serious public health problems. The MAR index results suggest that bacteria (*Proteus* spp., *S. aureus*, *P. aeruginosa*, *V. cholerae*, and *V. parahaemolyticus*) isolated from *O. niloticus* fish can contribute significantly to the spread of multidrug resistance and antibiotic resistance genes to consumers. The findings of the current study are in line with previous studies conducted in Nigeria (Chigor et al., 2020) and Malaysia (Noorlis et al., 2011; Saifedden et al., 2016).

The spread of antibiotic-resistant bacteria and antibiotic resistance genes is considered one of the most serious emerging threats to public health. An important factor in bacterial resistance to antimicrobials is that they carry related resistance genes (Iwu and Okoh, 2019; Iwu et al., 2020). To ascertain the resistant phenotypes, antibiotic-resistant gene profiles were conducted using the PCR method, which revealed the presence of resistance genes belonging to extended-spectrum β-lactamase, aminoglycoside resistance genes, aminoglycoside/streptomycin resistance genes, sulphonamide genes, and other β-lactamase resistance genes. There was a strong correlation between the phenotypic resistance pattern to the antibiotics for MDR bacteria and the presence of antibiotic resistance genes. However, only a few antibiotic resistance genes were selected for analysis in the study based on the antibiotic usage and previous reports from various regions of Kenya, although several types of antibiotics and their variants are used in the country. Therefore, the bacterial pathogens could contain other antibiotic resistance genes that were not included in this study. The need for routine surveillance/monitoring of potential pathogens in fish and fish products, including seafood in retail markets, remains a priority to ascertain the safety of these food products.

## Conclusion

Our results show high prevalence of antimicrobial-resistant foodborne pathogens (*Proteus* spp., *S. aureus*, *P. aeruginosa*, *V. cholerae*, and *V. parahaemolyticus*) in fish purchased from retail markets and underscore the risk associated with improper handling of fish. The MAR index results suggest that the bacteria (*Proteus* spp., *S. aureus*, *P. aeruginosa*, *V. cholerae*, and *V. parahaemolyticus*) isolated from fish can contribute significantly to the spread of multidrug resistance and antibiotic resistance genes to consumers. Also, the presence of antibiotic resistance genes in *Proteus* spp., *S. aureus*, *P. aeruginosa*, *V. cholerae*, and *V. parahaemolyticus* highlights the high risk to humans of exposure to resistant foodborne bacteria through consumption of undercooked fish.

Studies comparing the bacterial species composition from point of harvest to processed product, for example using tracer bacteria with particular characteristics (e.g., antimicrobial resistance markers), are needed to determine to what extent the bacterial flora of fish change during processing. It is also necessary for regulatory agencies in the country to increase the robustness with which they monitor and enforce the microbial safety of fresh *O. niloticus* fish and other fish products, and for vendors to strictly adhere to good food-handling practices. There is also a need to sensitize consumers to the need to ensure that they cook fresh *O. niloticus* fish well to allow for the removal of contaminating bacteria prior to consumption.

## Data availability statement

The datasets presented in this study can be found in online repositories. The names of the repository/repositories and accession number(s) can be found in the article/**Supplementary Material**.

## Ethics statement

Ethical review and approval was not required for the non-live fish samples because it is not needed in accordance with the local legislation and institutional requirements.

## Author contributions

MM, EN, JK, and EM conceptualized and designed the study. MM, EN, JK, EM, SM, HR, GM, BN, and JN contributed to methodolgy. MM and EN collected and analyzed data. EN and JN provided resources. MM prepared the first draft of the manuscript. MM, EN, JK, EM, SM, HR, GM, BN, and JN reviewed and edited the manuscript. All authors contributed to the article and approved the submitted version.
