# Supplementary material for: Prevalence and antimicrobial resistance profile of bacterial foodborne pathogens in Nile tilapia fish (Oreochromis niloticus) at points of retail sale in Nairobi, Kenya
Source: Front Antibiot. 2023 May 24;2:1156258. doi: 10.3389/frabi.2023.1156258 (PMC11731917; doi:10.3389/frabi.2023.1156258)
Supplement: Supplementary file 1 [file DataSheet_1.docx]

Supplementary Material

Prevalence and antimicrobial resistance profile of bacterial foodborne pathogens in Nile tilapia fish (*Oreochromis niloticus*) at the points of retail sale in Nairobi, Kenya

Millicent T. Mumbo^1,2,3^, Evans N. Nyaboga^2^, Johnson Kinyua^1^, Edward K. Muge^2^, Scholastica G. K. Mathenge^3^, GeoffreyMuriira^4^, Henry Rotich^4^, Bernard Njiraini^4^, and Joshua M. Njiru^4*^

^1^Department of Biochemistry, Jomo Kenyatta University of Agriculture and Technology, Nairobi, Kenya

^2^Department of Biochemistry, University of Nairobi, Nairobi, Kenya

^3^Department of Medical Laboratory Science, Kenyatta University, Nairobi, Kenya

^4^Research and development Department, Kenya Bureau of Standards, Nairobi, Kenya

***Correspondence**

Dr. Joshua Njiru
[njiruj@kebs.org](mailto:njiruj@kebs.org)

# Supplementary Tables

**Supplementary Table S1** Antibiotic resistance genes, primer sequences, expected amplicons sizes and PCR cycling conditions

| **Antibiotic resistance genes** | **Primer sequence (5′ → 3′)** | **Amplicons size (bp)** | **PCR cycling condition** | **Reference** |
| --- | --- | --- | --- | --- |
| ^bla^TEM-1 | F: TTG GGT GCA CGA GTGGGT  R: TAA TTG TTG CCG GGA AGC | 500 | 5 min initial denaturation at 94 °C followed by 35 cycles of 94 °C for 1 min, 57 °C for 1 min, 72 °C for 1 min and final extension at 72 °C for 10 min | Fang et al., 2008 |
| ^bla^CMY-2 | F: ATA ACC ACC CAG TCA CGC  R: CAG TAG CGA GAC TGC GCA | 600 | 5 min initial denaturation at 94 °C followed by 35 cycles of 94 °C for 1 min, 58 °C for 1 min, 72 °C for 1 min and final extension at 72 °C for 10 min | Fang et al., 2008 |
| *sul2* | F: CGG CAT CGT CAA CAT AAC C  R: GTG TGC GGA TGA AGT CAG | 722 | 5 min initial denaturation at 94 °C followed by 35 cycles of 94 °C for 1 min, 58 °C for 1 min, 72 °C for 1 min and final extension at 72 °C for 10 min | Falbo et al., 1999 |
| *tetA* | F: GCT ACA TCC TGC TTG CCT TC  R: CAT AGA TCG CCG TGA AGA GG | 280 | 5 min initial denaturation at 94 °C followed by 35 cycles of 94 °C for 1 min, 60 °C for 1 min, 72 °C for 1 min and final extension at 72 °C for 10 min | Ng et al., 2001 |
| *tetC* | F: CTT GAG AGC CTT CAA CCC AG  R: ATG GTC GTC ATC TAC CTG CC | 480 | 5 min initial denaturation at 94 °C followed by 35 cycles of 94 °C for 1 min, 62 °C for 1 min, 72 °C for 1 min and final extension at 72 °C for 10 min | Ng et al., 2001 |
| *dfrA7* | F: GGT AAT GGC CCT GAT ATC CC  R: TGT AGA TTT GAC CGC CAC C | 280 | 5 min initial denaturation at 94 °C followed by 35 cycles of 94 °C for 1 min, 58 °C for 1 min, 72 °C for 1 min and final extension at 72 °C for 10 min | Grape et al., 2007 |
| *strA* | F: CTT GGT GAT AAC GGC AAT TC  R: CCA ATC GCA GAT AGA AGG C | 600 | 5 min initial denaturation at 94 °C followed by 35 cycles of 94 °C for 1 min, 56 °C for 1 min, 72 °C for 1 min and final extension at 72 °C for 10 min | Velusamy  et al., 2007 |
| *aadA* | F: GTG GAT GGC GGC CTG AAG CC  R: AAT GCC CAG TCG GCA GCG | 500 | 5 min initial denaturation at 94 °C followed by 35 cycles of 94 °C for 40 s, 60 °C for 40 s, 72 °C for 40 s and final extension at 72 °C for 7 min | Velusamy  et al., 2007 |

**Supplementary Table S2:** Similarity of 16S rRNA sequences of MDR isolates of different bacteria pathogens from Nile tilapia, compared with that of accessions in the GenBank database.

| **No.** | **Isolate ID Code** | **Sub-County/Location of the isolate** | **16S rRNA Accession** | **Closest Match in Blast of the isolate** | **Similarity (%)** | **Accession of the closest match** |
| --- | --- | --- | --- | --- | --- | --- |
| 1 | MAK-38 | Makadara | OP293367.1 | *Proteus mirabilis* strain P13 | 98.32 | MT276300.1 |
| 2 | EMB-43 | Embakasi | OP293368.1 | *Proteus mirabilis* strain BLPS5 | 98.25 | ON460264.1 |
| 3 | LAN-46 | Lang’ata | OP293369.1 | *Proteus mirabilis* strain P13 | 98.87 | MT276300.1 |
| 4 | KS-19 | Kasarani | OP047935.1 | *Proteus penneri* strain A254 | 94.22 | KX692873.1 |
| 5 | KS-20 |  | OP047936.1 | *Proteus vulgaris* strain CQC01 | 94.89 | MN517893.1 |
| 6 | KS-23 | Kasarani | OP047937.1 | *Proteus penneri* strain njp2 | 96.89 | KU992679.1 |
| 7 | MK-24 | Makadara | OP047938.1 | *Proteus faecis* strain An Lec 78 | 96.58 | ON688699.1 |
| 8 | MK-25 | Makadara | OP047939.1 | *Proteus faecis* strain CA120921 | 96.23 | MG269475.1 |
| 9 | MK-27 | Makadara | OP047940.1 | *Proteus faecis*strain 08MAS2231 | 96.30 | MG269472.1 |
| 10 | MK-28 | Makadara | OP047941.1 | *Proteus alimentorum* strain NA-32 | 95.04 | MN882658.1 |
| 11 | EM30 | Embakasi | OP047943.1 | *Proteus alimentorum* strain NA-32 | 94.43 | MN882658.1 |
| 12 | EM33 | Embakasi | OP047945.1 | *Proteus penneri* strain njp2 | 98.51 | KU992679.1 |
| 13 | L-34 | Lang’ata | OP047946.1 | *Proteus faecis* strain 08MAS2231 | 96.97 | MG269472.1 |
| 14 | L-36 | Lang’ata | OP047947.1 | *Proteus penneri* strain njp2 | 96.80 | KU992679.1 |
| 15 | WT1 | Westlands | OP047928.1 | *Proteus faecis* strain TJ1636 | 96.75 | MG269469.1 |
| 16 | WT2 | Westlands | OP047929.1 | *Proteus* sp. SBP10 | 96.27 | GU812899.1 |
| 17 | WT7 | Westlands | OP047932.1 | *Proteus faecis* strain 08MAS1603 | 95.57 | MG269471.1 |
| 18 | WT8 | Westlands | OP047933.1 | *Proteus alimentorum* strain NA-32 | 96.42 | MN882658.1 |
| 19 | WES-03 | Westlands | OP293351.1 | *S. aureus* strain SA1 | 96.65 | OP364883.1 |
| 20 | WES-10 | Westlands | OP293352.1 | *S. aureus* strain SA1 | 96.17 | OP364883.1 |
| 21 | WES-11 | Westlands | OP293353.1 | *S. aureus* strain MRSA-4 | 97.17 | OP824648.1 |
| 22 | WES-13 | Westlands | OP293354.1 | *S. aureus* strain S1245 | 94.51 | KX447585.1 |
| 23 | KAS-15 | Kasarani | OP293355.1 | *S. aureus* strain EB12 | 96.99 | MT509600.1 |
| 24 | KAS-17 | Kasarani | OP293356.1 | *S. aureus* strain S1245 | 94.06 | KX447585.1 |
| 25 | KAS-18 | Kasarani | OP293357.1 | *S. aureus* strain RM_AST_SA001 | 94.05 | MK809238.1 |
| 26 | EMB-40 | Embakasi | OP293370.1 | *P. aeruginosa* strainBCr3 | 97.65 | KP717554.1 |
| 27 | EMB-41 | Embakasi | OP293371.1 | *P. aeruginosa* strain PB3A | 98.40 | KF029593.1 |
| 28 | EMB-42 | Embakasi | OP293372.1 | *P. aeruginosa* strain B13 | 97.59 | DQ350823.1 |
| 29 | WES-14 | Westlands | OP293360.1 | *V. cholera* strain W2-13 | 99.33 | KY496305.1 |
| 30 | KAS-21 | Kasarani | OP293361.1 | *V. cholera* strainCTI2 | 98.76 | KM362726.1 |
| 31 | WES-06 | Westlands | OP293358.1 | *V. parahemolyticus* strain TV18 | 92.62 | MT549167.1 |
| 32 | KAS-16 | Kasarani | OP293359.1 | *V. parahemolyticus* strainSR3 | 93.07 | KT006932.1 |

**Note:** The isolates L-39, L-44, WT-48, WT-49, KS-45 and KS-47 which were presumptive Proteus species were not included in the table and because their sequences showed no similarity with any isolate from GenBank database during BLASTn searches.

**Supplementary Figures**


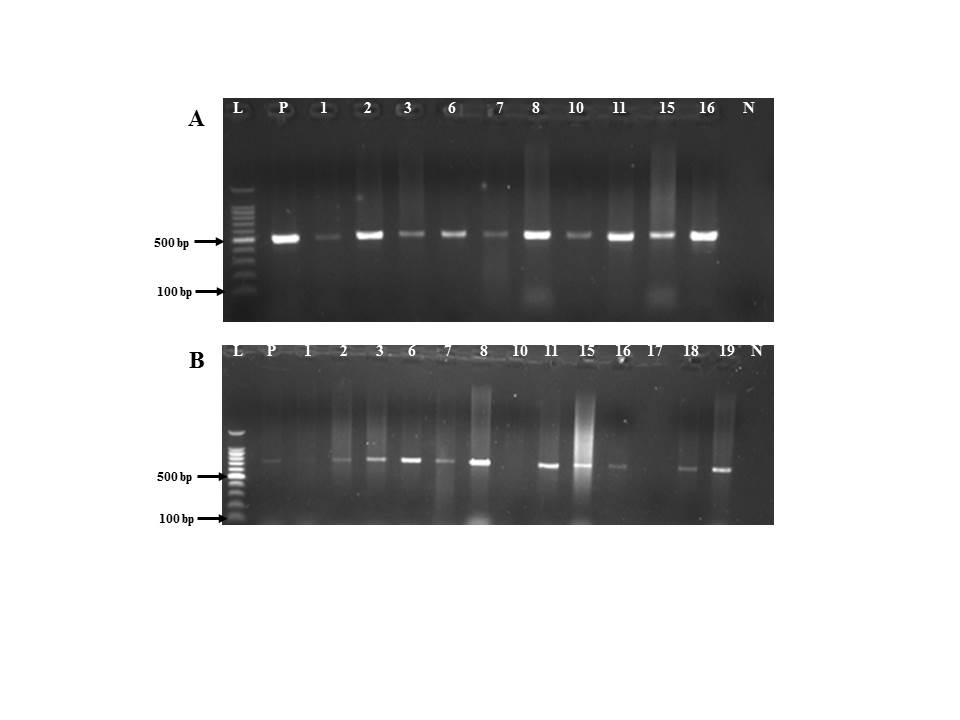


**Supplementary Figure S1** Agarose gel images showing amplification of antibiotic resistance genes (A) *bla*TEM-1 and (B) *sul2* for MDR isolates of different bacterial pathogens. Lane L: Molecular weight marker (100 bp), Lanes P and N represent positive and negative controls, respectively. Lane1 = *Proteus* sp. strain Wt1, 2 = *Proteus* sp. strain Wt2, 3 = *S. aureus* strain Wes-03, 6 = *V. parahemolyticus* strain Wes-06, 7 = *Proteus* sp. strain Wt7, 8 = *Proteus* sp. strain Wt8, 10 = *S. aureus* strain Wes-10, 11 = *S. aureus* strain Wes-11, 15 = *S. aureus* strain Kas-15, 16 = *V. parahemolyticus* strain Kas-16, 17 *= S. aureus* strain Kas-17, 18 = *S. aureus* strain Kas-18, 19 = *Proteus* sp. strain Ks-19.
